# A Comparison of Immunoglobulin Variable Region N-Linked Glycosylation in Healthy Donors, Autoimmune Disease and Lymphoma

**DOI:** 10.3389/fimmu.2020.00241

**Published:** 2020-02-18

**Authors:** Esther M. Vletter, Marvyn T. Koning, Hans Ulrich Scherer, Hendrik Veelken, Rene E. M. Toes

**Affiliations:** ^1^Department of Rheumatology, Leiden University Medical Center, Leiden, Netherlands; ^2^Department of Hematology, Leiden University Medical Center, Leiden, Netherlands

**Keywords:** glycosylation, autoimmunity, lymphoma, antibodies, B-cells

## Abstract

N-linked glycans play an important role in immunity. Although the role of N-linked glycans in the Fragment crystallizable (Fc) region of immunoglobulins has been thoroughly described, the function of N-linked glycans present in Ig-variable domains is only just being appreciated. Most of the N-linked glycans harbored by immunoglobulin variable domain are of the complex biantennary type and are found as a result of the presence of N-linked glycosylation that most often have been introduced by somatic hypermutation. Furthermore, these glycans are ubiquitously present on autoantibodies observed in some autoimmune diseases as well as certain B-cell lymphomas. For example, variable domain glycans are abundantly found by anti-citrullinated protein antibodies (ACPA) in rheumatoid arthritis (RA) as well as by the B-cell receptors of follicular lymphoma (FL). In FL, variable domain glycans are postulated to convey a selective advantage through interaction with lectins and/or microbiota, whereas the contribution of variable domain glycans on autoantibodies is not known. To aid the understanding how these seemingly comparable phenomena contribute to a variety of deranged B-responses in such different diseases this study summarizes the characteristics of ACPA and other auto-antibodies with FL and healthy donor immunoglobulins, to identify the commonalities and differences between variable domain glycans in autoimmune and malignant settings. Our finding indicate intriguing differences in variable domain glycan distribution, frequency and glycan composition in different conditions. These findings underline that variable domain glycosylation is a heterogeneous process that may lead to a number of pathogenic outcomes. Based on the current body of knowledge, we postulate three disease groups with distinct variable domain glycosylation patterns, which might correspond with distinct underlying pathogenic processes.

## Introduction

### Immunoglobulins Provide Humoral Immunity

B cell antigen receptors (BCR) are rearranged at the pre-B cell stage during B-cell development, and may be present as membrane-bound B-cell receptors or as soluble immunoglobulins. Through distinction between foreign and self-antigens, immunoglobulins play a key role in the defense against infections. Immunoglobulins can initiate a variety of inflammatory effects throughout the organism, depending on e.g., the recognized target, the isotype used or the properties of the Fragment crystallizable (Fc) tail. Once a B cell has encountered antigen, the B cell becomes activated and is able to secrete the B-cell receptor, particularly after maturation to antibody-secreting plasma blasts and plasma cells, in soluble form as IgM. Upon further activation and acquisition of T cell-help, class switching from IgM to IgG, IgA, or IgE can occur, leading to the presence of antigen-specific, isotype-switched antibodies in serum and other bodily fluids (1). Of all immunoglobulins, IgG is the most prominent in serum, with a concentration of ~10 mg/mL (2, 3). IgG directly links the innate to the adaptive immune system by activating the complement system and binding to Fc receptors. Likewise, it can also mediate uptake of microbes by dendritic cells and macrophages, which transport the pathogen to secondary lymphoid organs for further initiation and activation of the adaptive immune response. Likewise, immunoglobulins can facilitate antibody-dependent cell mediated cytotoxicity (ADCC), a process in which FcγR-receptor activation leads to pathogen lysis by natural killer cells (4, 5). Furthermore, complement-dependent cytotoxicity (CDC) enhances cellular pathogen uptake for subsequent antigen presentation (5).

### Addition of Glycans Shapes Immunoglobulin Function

**Figure 1:**
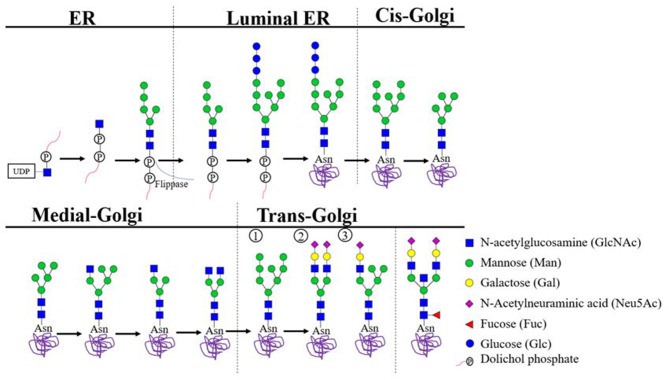
Biosynthesis of N-linked Glycans. In the cytoplasm of the endoplasmic reticulum (ER), GlcNAc-P combines with dolichol phosphate to generate dolichol pyrophosphate N-acetylglucosamine (Dol-P-P-GlcNAc). Stepwise addition of another N-acetylglucosamine, nine mannose and three glucose residues results in the precursor complex. Which is co-translationally transferred *en bloc* to an eligible asparagine residue located on the luminal side of the ER. Eligibility depends on the presence of the consensus glycosylation motif (N-X-S/T X≠P) and position of the asparagine in the 3D protein structure. Subsequent trimming steps result in glycans carrying eight or nine mannose residues, which proceed to the cis-Golgi complex to undergo additional trimming. In the medial Golgi, further biosynthesis may trim mannose residues and/or attach N-acetylglucosamine residues. Ultimately, three core types of glycans may be distinguished: (1) uncleaved terminating mannoses result in a high mannose type glycan; (2) at least two terminal N-acetylglucosamine residues are further extended to create a complex type glycan; (3) a single terminal N-acetylglucosamine residue results in a hybrid-type glycan. All glycan types may gain a fucose residues attached to the glycan core. Additionally, both the hybrid and the complex-type glycans may become resistant to further trimming through addition of a bisecting N-acetylglucosamine residue to the glycan core. Hybrid and complex type glycans may gain prolonged antennae with additional N-acetylglucosamine or sulfate. Such α-linked capping sugars readily interact with antibodies and lectins (6–10).

Harboring of N-linked glycans is a result of the co-translational covalent addition of carbohydrate groups to asparagine residues in the lumen of the endoplasmic reticulum (ER). This process is primed by the presence of N-linked glycosylation motifs, which consist of an asparagine (N), followed by any amino acid but proline, followed by serine (S) or threonine (T) (N-X-S/T; X≠P). Initially, a pre-formed lipid-linked glycan consisting of two N-acetlyglucosamines (GlcNAc) linked to nine mannose (Man) and three glucose (Glc) residues is attached *en bloc* (dolichol-P-P-GlcNAc_2_Man_9_Glc_3_). When the protein is subsequently transferred to the Golgi complex, glycosyl-hydrolases and transferases further diversity the attached glycan (Figure 1) (11). Finally, three main types of glycans can be identified (Figure 1) (12). First, a high mannose-type glycan when the terminating mannoses remain uncleaved. Second, a “complex-type glycan” which can be heterogenous and is found in over 30 different species on antibodies (Table 1) (12). Complex glycans consist of at least two “antennae” formed by presence of N-acetylglucosamine residues to the mannose core, but can also express three-or four antennae (12). These antennae can be extended through the addition of galactoses and terminal sialic acid. Furthermore, complex glycans can also contain a bisecting N-acetylglucosamine and a core fucose. The latter sugar moiety, when expressed by Fc-glycans of IgG, can mediate a strong impact on FcγR-binding (26). Third, a hybrid-type glycan which has one single terminal N-acetylglucosamine residue to which galactose and then sialic acid can be attached and one arm with mannoses (12).

**Table 1:** Overview of all the diseases mentioned and their N-linked glycan specifications.

	**Frequency**	**Distribution**	**Glycan type**	**Proposed function**
HD	15–20% (13)	CDR3	Complex (bisecting GlcNAc, sialylated) (14, 15)	
RA ACPA	>90% (13)	FR3 (16)	Complex (highly sialylated, bisecting GlcNAc, core fucose) (13)	Creates possible optimal balance
ANCA MPO	Unknown	FR3 (17)	Complex (galactosylated and mono-or di-sialylated) (18)	Impact functional and structural characteristics of the immunoglobulin
SLE	Unknown	Unknown	Unknown	
pSS anti-SS-A and/or anti-SS-B	~23% (19, 20)	FR3 (20)	Complex (20) (Sialylated)	Selection advantage
MG anti-MuSK	Unknown	Unknown	Unknown	
IgG4-RD	Unknown	Unknown	Complex (Sialylated) (21)	Unique effector function, interaction with Siglecs
FL	79–100% (22)	CDR2 (22, 23)	Mannose	Selection advantage
PCFCL	Unknown	CDR2 (24)	Unknown	
DCBCL	41% (22)	CDR3 (25)	Unknown	
BL	82% (22)	Unknown	Unknown	

*HD, Healthy Donor; CDR3, Complementarity-Determining Region 3; RA, Rheumatoid Arthritis; FR3, Frame Work 3; ANCA MPO, Anti-Neutrophil Cytoplasmic Autoantibody (ANCA) Associated Vasculitis (AAV), myeloperoxidase; SLE, Systemic Lupus Erythematosus; pSS, primary Sjogren's Syndrome; SS-A, Anti-Sjögren's-syndrome-related antigen A; SS-B, Anti-Sjögrens's-syndrome-related antigen B; MG, Myasthenia Gravis; MuSK, muscle-specific kinases; IgG4-RD, Immunoglobulin 4 Related Disease; FL, Follicular Lymphoma; CDR2, Complementarity-Determining Region 2; PCFCL, Primary Cutaneous Follicle Center cell Lymphoma; DCBCL, Diffuse large B-cell lymphoma; BL, Burkitt's Lymphoma*.

### Fc Glycosylation

Immunoglobulins are glycoproteins with conserved N-linked glycans in their Fc tail. For example, all IgG subclasses harbor N-linked glycans at heavy chain position N297 (27). It is becoming increasingly evident that these Fc-glycans can have major immunoregulatory functions (28, 29). For example, IgE Fc-glycosylation has been shown to play a role in allergic reactions by modulating the interaction of IgE to its Fc Receptor (30). Fc-glycan variability is relatively limited by the three-dimensional structure of the protein and peptide-glycan interactions. In addition, the limited accessibility of glycosyltransferases to the glycan mostly prevents extension of the glycan by sialylation and galactosylation (31, 32). Nevertheless, the Fc-glycans still vary greatly in composition and around 30 different glycan structures were observed for IgG, the majority of which are complex-type biantennary structures (33).

### Variable Domain Glycosylation

In contrast to the ubiquitous N-linked glycosylation of the Fc-tail, only a proportion of immunoglobulins contains additional N-linked glycans in the variable domain. In the naïve human B-cell repertoire, N-linked glycosylation motifs are only present in the variable domain when rearrangements contain the V-segments IGHV1-8, IGHV4-34, or the more rarely used IGHV5-10, IGKV5-2, IGLV3-12, and IGLV3-22 (34). Additional N-linked glycosylation motifs may be found in naïve B cells when junctional diversity creates novel motifs in the complementary-determining region 3 (CDR3). Whereas, the influence of conserved N-linked glycans on the Fc tail effector functions is becoming increasingly well-understood (13, 21, 35–37), N-linked glycosylation of the antigen-binding fragment (Fab) arms, responsible for antigen binding, has been less studied (13, 29). As a consequence, the mechanisms and functions of variable domain glycosylation are less well-understood as compared to Fc glycosylation for which the immune-related impact and immune modulatory effects are well defined (38–41). For example, different IgG Fc-glycoforms can influence Fcγ-Receptor binding, and thereby control the activation of e.g., FcγRIII-expressing immune cells by immune complexes (42). Some Fc-glycoforms, expressing high levels of sialic acid-residues, have even described to display anti-inflammatory effects, thereby contribution to the control of inflammation and inflammatory processes (26, 43). Nevertheless, it is highly conceivable that also variable domain glycosylation can have an impact on immune function since it is abundantly present in both autoimmune diseases and certain B-cell lymphomas (18–20, 22, 24, 44–47).

### General Glycan Composition

The type of glycans present in the Fc-part and the Fab-part can differ considerably. Fc glycans show increased degrees of fucosylation as compared to variable domain glycans. Conversely, variable domain glycans are more often galactosylated, bisected, and sialylated, putatively due to the Fc glycan being shielded in between two heavy chains in the quaternary immunoglobulin protein structure, decreasing its accessibility for the glycosyltransferases responsible for the complex glycan structures (14). In contrast, the variable domain of antibodies is more likely to be exposed when traversing the Golgi complex, leading to more fully maturated glycans. However, due to the inherently diverse nature of the variable domain, local changes due to differential VDJ usage and somatic hypermutation may result in varying local differences to enzyme accessibility (38). Furthermore, the influence of disease and physiological factors such as hormones, age, cytokine release, and pregnancy, may induce an altered expression of glycosyltransferases in B cells, skewing the newly attached structures toward certain glycoforms in both the variable domain and the Fc-region alike (38, 48–50).

### Variable Domain Glycosylation in Healthy Donors

#### Prevalence

Variable domain glycans have been estimated to be present in 15–20% of IgG immunoglobulins (combining heavy and light chain prevalence) in healthy individuals. In general, estimates from genetic analyses have been slightly lower than from protein-based strategies, such as the measurement of sialic acids to determine the presence of variable domain glycans (13, 29, 51–54). One explanation for this discrepancy, is offered by the observation that the stability of secreted immunoglobulin may be positively affected by the presence of variable domain glycans acquired during antigen-specific immune responses (thereby also potentially offering an *in vivo* selection mechanism for these antibodies) (55). As this enhanced stability would lead to an increased half-life, variable domain glycosylated serum immunoglobulins may be relatively abundant compared to their prevalence in the cellular compartment.

#### V Allele Distribution

Variable domain glycans were found to cluster around antigen-binding sites, suggesting that they are not randomly acquired, but rather lead to selective outgrowth of B cells (24, 53). A possible explanation is that the addition of these glycans induce a significant change in antigen affinity and binding, thereby modulating the interaction between antigen and antibody (53).

#### Glycan Composition

Over 90% of healthy donor IgG N-linked glycans contain a core fucose, of which the fucosylated digalactosylated form is the most abundant (14, 56). The majority of the N-linked glycans found on IgG plasma are of the complex biantennary type which are characterized by a high degree of conformational flexibility. In comparison with Fc glycans, the IgG variable domain glycans contain low percentages fucose and high percentages of sialic acids, bisecting GlcNAc and galactoses (15, 18, 57), with some differences between individuals depending on circumstances such as age and pregnancy (57). The high mannose type glycan is rare in healthy donor variable domains, estimated to represent only 4% of IgG variable domain glycans (38).

#### Subclass-Specific Differences

Within immunoglobulins, there are key differences found between subclasses. Recently, both IgE and IgG4 were shown to contain significantly more variable domain glycans than other isotypes (46, 54, 58). In particular, bone marrow-derived IgE was found to contain twice as many variable domain glycans as other immunoglobulin subclasses in healthy donors (46), despite the fact that they often carried fewer mutations. IgE VDJ often retained germline-encoded N-linked glycosylation motifs and acquired additional ones at a faster pace than IgG and IgA. It has been suggested that in this way, variable domain glycosylation blocks antigen recognition sites and thereby creates a low-affinity resident IgE repertoire, which could protect against allergic reaction by occupation of Fcε receptors (54). Similarly, IgG4 contained more N-linked glycosylation motifs than IgG1-3. Indeed, the number of N-linked glycosylation motifs was similar to that found in IgE (46), which could suggest a commonality as both subclasses are known to be involved in T-helper 2 type responses.

### Variable Domain Glycosylation in Autoimmune Diseases

Autoimmune diseases are characterized by an adaptive immune response against host tissues. A growing body of evidence shows an increased prevalence of autoantibody variable domain glycosylation in the context of many autoimmune diseases (59).

#### Immunoglobulin G4-Related Disease

In light of the differential variable domain glycosylation of immunoglobulin subclasses in healthy donors, immunoglobulin G4-related disease (IgG4-RD) represents a particularly interesting disease, as it is characterized by increased IgG4 titers. IgG4-RD is a multisystem fibroinflammatory condition, in which tumor-like masses related to organ dysfunction and tissue infiltration by IgG4^+^ plasma cells may be found in a number of anatomical locations, particularly the bile ducts (60, 61). Recently, variable domain glycosylation was found to be increased in IgG4-RD patients in comparison to healthy controls and patients with non-IgG4-related primary sclerosing cholangitis (21). In particular, as detected by Sambucus nigra agglutinin (SNA) affinity chromatography, the presence of variable domain sialylation was increased in both IgG1 and IgG4 subclasses, even when corrected for the inherently higher N-linked glycosylation rates in IgG4 variable regions (46). This observation was corroborated by another study investigating combined variable domain and Fc-glycosylation in IgG4-RD patients (44). Although the role of increased variable domain glycosylation is unclear, it is suggested that increased variable domain sialylation could impart IgG4 immunoglobulins in the context of IgG4-RD with disease-specific immunomodulatory properties through binding of SIGLECs such as CD22 (62).

#### Rheumatoid Arthritis

Rheumatoid Arthritis (RA) is a destructive, autoantibody-mediated inflammatory disease of the joints which affects ~0.5–1% of the population (63, 64). The anti-citrullinated protein antibodies (ACPA), that are typically found in RA patients, target proteins that have undergone a post-translational conversion from arginine to citrulline (65). ACPA have been shown to bind their antigens with low affinity (66), and can occur in different isotype compartments including IgG, IgM, or IgA (67).

We were the first to observe the abundant presence of N-linked glycans in the variable domain of ACPA-IgG (45) and provided first evidence indicating that these glycans are introduced during somatic hypermutation and thereby could, potentially, influence the binding to citrullinated antigens. As previously mentioned, healthy control IgG contains 15–20% variable domain glycans, whereas this percentage is considerably increased in ACPA-IgG to over 90% (13). It has been postulated that these glycans modulate the antibody's interaction with citrulline. ACPA acquire this feature during affinity maturation as a consequence of somatic hypermutation. Indeed, for ACPA-IgM, no increase in variable domain glycosylation was observed (13). The latter observation is in line with the notion that T-helper cell activity is required for the generation of N-linked glycosylation-sites and hence the addition of variable domain glycans.

The ACPA-IgG variable domain glycans show high degrees of sialylation, could impart an immune-modulatory function (13, 68, 69). If this is the case, ACPA could locally shape immunological microenvironments at inflamed diseases sites, as synovial fluid-derived ACPA exhibited higher variable domain glycosylation prevalence than ACPA obtained from peripheral blood (13).

#### Anti-neutrophil Cytoplasmic Autoantibody

Anti-neutrophil cytoplasmic autoantibody (ANCA) associated vasculitis (AAV), is characterized by inflammatory lesions of the blood vessels (70, 71). Of the two predominantly recognized antigens, proteinase 3 (PR3) and myeloperoxidase (MPO) (72, 73), only the latter has been associated with increased levels of sialylated variable domain glycans (17). The recent description of SHM introduced glycosylation motifs, confirmed observations of increased variable domain glycan prevalence in AAV (17, 18). As with other autoimmune diseases, the function and implication of these autoantibodies are as yet incompletely clarified (18).

#### Systemic Lupus Erythematosus

Systemic Lupus Erythematosus (SLE) is an autoantibody-mediated chronic disease characterized by multi-organ involvement that typically affects women (74, 75). In most cases of SLE, high titers of antinuclear antibodies (ANA) directed at nuclear and cytoplasmic cell components may be detected, and thus prove useful as a diagnostic marker (76, 77). Recently, it was observed that patients with SLE showed a 6% increase in the amount of acquired N-linked glycosylation motifs in the variable domain compared to a control data set (19). However, if variable domain glycosylation is truly enhanced on mature proteins, and whether it plays a role in SLE pathogenesis and/or disease activity or is rather a bystander-effect, needs yet to be confirmed.

#### Primary Sjogren's Syndrome

Primary Sjogren's Syndrome (pSS), is characterized by lymphocytic infiltrates in the exocrine glands (78). A hallmark of this disease is the presence of anti-RO (SS-A) and anti-La (SS-B) antibodies (79). Increased IgG variable domain glycosylation has been observed in pSS patients as compared to healthy controls (20). The authors noted an absence of evidence for antigen selection pressure in the variable region sequences, and proposed that antigen-independent interaction of the variable domain glycans with parotid gland microenvironmental lectins might represent an alternative stimulus for B-cell proliferation (20).

#### Myasthenia Gravis

In Myasthenia Gravis (MG), autoantibodies against neuromuscular junction protein impair neuromuscular transmission, resulting in skeletal muscle weakness (80). Although MG is typically characterized by antibodies against the acetylcholine receptor (81, 82), in rare cases the pathologic mechanisms derives from antibodies directed against muscle-specific kinases (MuSK) (83). These MuSK autoantibodies are present in 5–10% of the patients (80, 84) and are typically of the IgG4 subclass which are associated with anti-inflammatory responses. To a lesser extent, IgG1 anti-MuSK antibodies may be found alongside the IgG4 variants (85). To our knowledge, Koers et al. were the first to investigate the frequency and distribution of variable domain glycans in MG patients (46). In these studies, no differences were found in the distribution of the glycosylation motifs compared to healthy controls (46). However, recently variable domain glycans have been observed in monoclonal anti-MuSK-antibodies, although the phenomenon was not ubiquitous in this disease, leading the authors to suggest that variable domain glycosylation is not essential for the generation of anti-MuSK antibodies (85). Since it was also observed that the characteristics of bulk memory B cells in MuSK MG patient do not differ from healthy controls (46), the conclusion that variable domain glycosylation plays no major role in MuSK MG seems warranted. However, such conclusions should be corroborated by *in vivo* observations rather than genetic analyses, as IgG4 may undergo Fab-arm exchange with the potential of radically changing the antibody's properties (86).

In summary, variable domain glycans are present in high prevalence in a number of autoimmune diseases with disease-specific autoantibodies. For some (such as RA and AAV), the ubiquitous presence of variable domain glycans, suggest a mechanistic role, for example in the breach of immunological tolerance of variable domain glycans in these diseases. In other autoimmune diseases, variable domain glycosylation is less abundant (e.g., SLE, MG, and pSS). The reason for these differences is not known, but might relate to the type of antigens recognized or the chronicity/abundance of antigen-exposure. In comparison with healthy donor IgG, auto-antigen IgG from patients with autoimmune diseases can show an enhanced variable domain glycosylation (13). Nonetheless, the glycan composition of the overall IgG response is similar as in both situations complex-type glycans are observed, although an elevated level of highly sialylated glycan-species have been on auto-antigen-specific IgG in RA (13, 15, 18).

### Variable Domain Glycosylation in B-Cell Malignancies

Intriguingly, the abundant presence of variable domain glycans has not only been observed in autoimmune disease, but also in the case of certain lymphomas. These findings are interesting as both disease groups may result from poor immune regulation and could therefore point to an unappreciated shared pathogenic mechanism.

#### Follicular Lymphoma

Follicular lymphoma (FL) is the most common indolent B-cell lymphoma, representing roughly 40% of all non-Hodgkin lymphomas (22). Immortalized by BCL-2 overexpression, FL cells remain situated in the germinal center stage leading to ongoing somatic hypermutation after malignant transformation (87). Already two decades ago, it was observed that FL BCR commonly feature SHM-induced N-linked glycosylation motifs (22). Indeed, such motifs are present in 79–100% of FL, and seemingly cluster around “hotspot” sites at positions 38 (CDR1), 55 (FR2), 107 (CDR3), and 125 (FR4) on the FL BCR (24). Such a ubiquitous presence of acquired glycans suggests a key role in lymphomagenesis. A popular theory involves glycan interaction with microenvironmental lectins, or alternatively with lectins from opportunistic bacteria. This interaction would stimulate the BCR via exposed variable domain glycans and in doing so provide additional growth and survival signals to the lymphoma cell (40, 88–90). An interesting and unusual finding on N-linked glycans found on FL B cells is that they appear to be rich in the otherwise rare oligomannoses in the variable regions, even though they feature complex-type glycans in the Fc domain (91, 92).

#### Primary Cutaneous Follicle Center Cell Lymphoma

Primary Cutaneous Follicle Center Cell Lymphoma (PCFCL) is an uncommon indolent B-cell lymphoma of unknown etiology that is found preferentially on the skin of the scalp and trunk (93). As to immunophenotyping and morphology, PCFCL mirrors FL (94, 95). Indeed, similar to FL it was recently observed that the vast majority of PCFCL BCR feature up to four acquired variable domain glycans (24). Unlike FL however, there was no evidence of ongoing SHM, suggesting that the variable domain glycans must have been present already at malignant transformation. Although the majority of the acquired N-linked glycosylation motifs in PCFCL were found in the CDRs, the exact positions differ between the two diseases, with PCFCL showing no proclivity to cluster around hotspots. Whether these N-glycosylation motifs indeed lead to *in vivo* glycosylation, remains to be confirmed (24).

#### Burkitt's Lymphoma

Burkitt's lymphoma (BL) is an aggressive B-cell lymphoma associated with Epstein Barr virus and Human immunodeficiency virus which accounts for ~1–5% of the non-Hodgkin lymphomas (96). Like FL, BL may incidentally show intraclonal sequence variation (47, 97), although the true extent of this phenomenon has, at least to our knowledge, yet to be verified. Many BL, especially the endemic variant, acquire novel glycosylation sites (47). Additionally, many BL BCR which have not acquired novel variable domain glycans, have been found to express a rearranged IGHV4-34 gene which contains a germline-encoded variable domain glycan. Some cases lost their germline sites, but 75% of these had acquired a newly formed site. This implies that there is a selective pressure to maintain at least one variable domain glycan, although how this influences BL disease biology is currently unclear, as no functional experiments as with FL have been performed.

#### Diffuse Large B-Cell Lymphoma

Diffuse large B-cell lymphoma (DCBCL) is a common lymphoid malignancy and accounts for 40% of non-Hodgkin lymphoma. DLBCL is clinically heterogenous, but generally aggressive and characterized by the presence of mature B cells (98). One study showed variable domain glycosylation in up to 41% of DLBCL (22). However, since all DLBCL were included, this may represent an overestimation of primary DLBCL, as a subset might represent cases of the ubiquitously glycosylated FL which have undergone histological transformation. Therefore, it remains unclear whether variable domain glycans are found in an increased prevalence in primary DLBCL, as the relatively small datasets used do not provide sufficient evidence to support these observations (24).

#### Variable Domain Glycans May Serve to Distinguish Lymphoma Types

Summarizing, B-cell lymphomas with a follicular growth pattern have a strong proclivity to acquire variable domain glycans. Indeed, the variable domain glycans may in fact determine their follicular architecture as it links the cell to the germinal center microenvironment (22). Since normal plasma cells, memory B cells, multiple myeloma cells and chronic lymphocytic leukemia cells do not show a marked increase in N-linked glycosylation motifs, it appears that variable domain glycans are of less importance to cells that have exited the germinal center. Moreover, it appears that the mere presence of any variable domain glycan is not sufficient, but N-linked glycosylation motifs need to occur in specific locations, as witnessed by the FL hotspots and the fact that naturally occurring IGHV4-34 variable domain glycans are often lost and replaced with novel motifs. Given these findings, antigen-independent BCR activation through interactions between glycans and microenvironmental lectins could represent a main driving mechanism for lymphomagenesis (89, 90).

### A Comparison of Physiology, Autoimmune Diseases and Lymphoma

#### Distribution of the N-Linked Glycosylation Motifs

**Figure 2:**
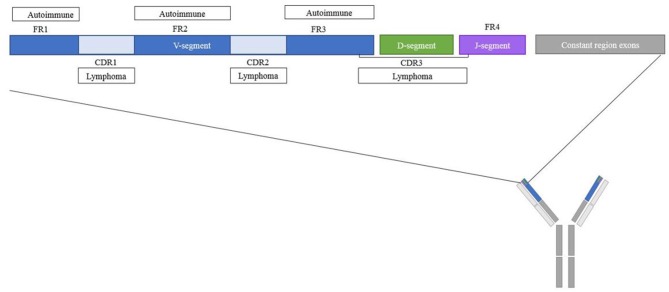
Schematic overview of regions where N-linked glycosylation motifs are found for different diseases. Overview of the location within the variable domain of frequently found glycosylation motifs for autoimmune diseases and lymphomas. The majority of the variable domain glycosylation motifs in autoimmune diseases were found in the CDR regions, for lymphomas the majority of the variable domain glycosylation motifs were found in the FR regions.

The position of an N-linked glycosylation motif in the variable region may prove informative for its functional consequences. Indeed, depending on the variable region position, glycans may block antigen-binding, contribute to antigen-specificity or interact with other molecules without interfering with the antigen-binding site. An analysis of structural regions containing the newly acquired glycans may help to make a rough distinction between these possibilities. The majority of the N-linked glycosylation motifs in patients with pSS, RA and AAV were observed in framework region 3 (FR3) (Figure 2, Table 1) (16, 17, 20). In addition, RA patients' ACPA showed a slight increase in the amount of glycosylation motifs in CDR1 and a slight decrease in CDR3 compared to healthy donors (16). This finding could be interesting since FR3 is known for its interaction with super-antigens which are known to stimulate B-cell differentiation and antibody secretion (42). The tendency of the N-linked glycosylation motifs to appear in the FR could even represent an alternative pathway for autoreactive B cells generation independent of classic antigenic selection (19). Indeed, less of such enrichment is thus far observed in healthy donors, for whom the majority of the glycosylation motifs were located in the CDR regions (16, 46, 53). Future determination of variable domain glycan clustering in IgG4-RD, SLE, and MG may provide additional evidence toward this hypothesis.

In contrast to acquired variable domain glycans in autoimmune diseases, 87% of heavy chain variable domain glycosylation motifs of FL patients were observed in the CDR. Moreover, rather than clustering on particular regions, FL BCR acquired N-linked glycosylation motifs in a number of very specific hotspots (24), with the strongest hotspot located in CDR2 (Figure 2, Table 1) (22, 23). These latter sites were introduced during somatic hypermutation by introduction of an asparagine residue, which is an uncommon event for healthy B cells or CLL cells at this position (22). Likewise, acquired PCFCL variable domain glycans can often be found in the CDR2 region, although they do not cluster around the canonical FL hotspots (24). DLBCL variable domain glycans appear to cluster around the CDR3 region (25), and SHM-acquired BL variable domain-glycans also favor the CDRs, especially CDR2 (47, 99). Some authors have suggested super-antigens as a driver of clonal selection and expansion of variable domain glycosylated B cells in BL (99).

These differences in distribution indicate an interesting difference in variable domain glycosylation between lymphoma and autoimmune disease. The variable domain glycans typically found in lymphoma CDR, could prove to be detrimental to autoantibodies. Indeed since the CDR is intimately involved in antigen binding, blocking the antigen-binding site with a large glycan molecule may be expected to interfere with autoantibody recognition, a requirement for B-cell growth and selection. Conversely, the strong hotspot clustering of variable domain glycans in lymphoma BCR could also be explained as a result of glycans contributing to or “mimicking” antigen-binding, especially when those antigens are environmental or bacterial lectins. Whether those antigens are indeed lectins, other microenvironmental antigens or the substrate of physiological B-cell activity before malignant transformation, is one of the key open questions to understand the importance of this phenomenon. Alternatively, the hotspot clustering may not signify antigen-recognition at all, but could contribute to auto-stimulatory properties due to tertiary formations of the BCR [as described by a different process in for example CLL (100)].

#### Composition of the Glycan

Although there is generally a decreased amount of Fc sialylation and galactosylation in autoimmune diseases (101, 102), *N*-linked sugars present on ACPA IgG variable domains form a notable exception with their high sialic acid content of up to 44% (13). Similarly, increased sialylation of variable domain glycans was, as compared to healthy controls also recently observed in the IgG variable domain glycans from patients with AAV and IgG4-RD, the latter regardless of Fab-arm exchange (18, 21). To underline the disease-specificity of this phenomenon, it is worth noting that even subgroups of patients with the same diseases, such as high-activity ANCA-PR3, have been reported to express strikingly low levels of variable domain sialylation. Nonetheless, variable domain sialylation may prove to be a common hallmark of autoantibodies in a plethora of autoimmune diseases (Table 1). Since sialic acids are so prominently present on variable domain glycans in ACPA B cells, it might be possible that they give these B cells a survival advantage compared to B cells without the sialic acids.

In contrast to the highly sialylated variable domain glycans in RA, AAV, and IgG4-RD patients, FL variable domain glycans were reported to be rich in high-mannose structures (Table 1) (91, 92, 103). Such glycans were solely observed at sites which were newly acquired through somatic hypermutation. In 85% of FL cases, non-germline variable domain glycans were found in the CDR, of which the majority were high-mannose structures (91). Their near-universal presence in this disease, may signify that they are beneficial or even essential to improve proliferation and survival (40). In healthy donors, these high mannose structures are normally not found on the cell surface, sparking the hypothesis that in FL the glycans do not fully mature inside the Golgi complex due to enzyme inaccessibility (91, 92).

#### Glycan-Lectin Interaction

The variable domain glycans of autoantibodies are, by and large, located at different positions as in FL and the mechanism by which the variable domain glycans of autoantibodies convey a survival advantage does not immediately become clear. However, it may be well-explained by interaction of highly sialylated variable domain glycans with sialic acid-binding immunoglobulin-type lectins (SIGLECs). Through interaction of sialic acids with for example SIGLEC CD22, an inhibitory co-receptor of the B-cell receptor (104), an optimal balance between stimulatory and inhibitory signals is achieved. By interfering with e.g., CD22 or other cellular lectins, the threshold for activation of ACPA-expressing B cells may be modified, leading to a selective advantage to those ACPA-expressing B cells that have introduced an N-linked glycosylation site. In case such interactions are underlying the apparent selective advantage mediated by variable domain glycans to autoreactive B cells, it might explain the differential localization of these glycans in autoimmunity vs. FL.

As mentioned above, in case of FL, it has been proposed that interaction with environmental lectins act as a driving force behind the formation of variable domain glycans. In this case, glycan-lectin-interaction does not modulate or disrupt an inhibitory receptor, but rather serves as surrogate to mediate BCR-crosslinking. Indeed, it was shown that lectins can bind variable domain glycans introduced during somatic hypermutation, and in this way provide a survival signal for autoreactive B cells from the germinal center and onwards during development (88). Furthermore, experimental models confirmed interaction between lectins and FL high-mannose variable domain glycans, thereby stimulating the cells (40, 88). FL variable domain glycans have been reported to interact with C-type lectins, for example dendritic cell-specific intercellular adhesion molecule-3-grabbing non-integrin (DC-SIGN) (40). DC-SIGN is expressed by dendritic cells and macrophages, both of which may be abundantly found in FL biopsies. DC-SIGN binding was shown to trigger BCR signaling (88–90), but so did other lectins, such as those on opportunistic bacteria (40). In ultimo, it was hypothesized that lymphomagenesis through the latter pathway could be interrupted by targeted antibiotic treatment (24). However, more characterization of both the auto-lectins and lectins from opportunistic bacteria would be necessary to fully understand the pathogenesis of FL and create possible treatment options. Since PCFCL shares a few characteristics with FL, it is thought that the variable domain glycans of PCFCL patients are also mannosylated, potentially leading to stimulation from lectins from the cutaneous microbiome (24). Only little is known about the functionality of BL and DLBCL variable domain glycans, but it is possible that lectin interactions also plays a role in BCR activation in these diseases. Although a similar mechanism may mediate disease activity in variable domain glycosylated autoantibodies, we are unaware of any published experimental evidence to support or deny this possibility.

## Conclusion

Concluding, variable domain glycans appear to play an important role in several B-cell diseases, both hemato-oncological and autoimmune in nature. However, in both fields the precise role and consequences of the variable domain glycosylation remain at best incompletely understood. There are, nonetheless, some observed commonalities between N-linked glycans in autoimmunity and in hematology. Lectin interaction is a recurring hypothesis in both autoimmune and malignant settings, although additional evidence is warranted to further support these paradigms.

Careful scrutiny of the distribution of variable domain glycans could hold the key to an increased understanding of any of these diseases. Currently, only RA ACPA and FL BCR have been investigated in extensive detail, which has led to corresponding pathogenic postulations of the variable domain glycans. Researchers should aim to compile publicly accessible compendia for all other implied or potentially affected diseases, so side-by-side comparison may clarify important commonalities and differences for each disease.

A possible lead to further understanding of the contribution of variable domain glycans in different disease, is the different distribution and composition of variable domain glycans in autoimmunity and malignant hematological disease. Such differences suggest that in these disease groups, whilst both apparently resulting from deficient immune monitoring, variable domain glycosylation may play distinctly different roles.

On the other hand, defining common variable domain glycosylation patterns should help cluster diseases by shared underlying pathological mechanisms. Based in the current body of knowledge, we propose three such clusters: (1) near ubiquitous CDR-glycosylation with high-mannose structures, which is typical for certain lymphoma types and may depend on auto-lectin interactions potentially mimicking BCR-triggering; (2) near-ubiquitous FR(3)-glycosylation with extensive sialylation that may facilitate SIGLEC interaction, comprising some well-studied autoimmune diseases; and (3) Increased, but non-ubiquitous (FR) glycosylation in autoimmune diseases, which may well be a result of concurrent immune processes rather than an independent driving mechanism.

All in all, variable glycosylation in B-cell diseases is an emerging field of investigation. Through comparison of both commonalities and differences in variable glycosylation of B-cell diseases across a number of fields, diseases may be clustered by shared pathogenesis and currently missing causative mechanisms may be clarified.

## Author Contributions

EV and MK screened, summarized and analyzed available literature, and wrote the manuscript. MK, HS, HV, and RT provided project supervision. All authors have critically read and approved the manuscript.

### Conflict of Interest

The authors declare that the research was conducted in the absence of any commercial or financial relationships that could be construed as a potential conflict of interest.
